# Limited impact of hepatitis A virus 3C protease-mediated cleavage on the functions of NEMO in human hepatocytes

**DOI:** 10.1128/jvi.02264-24

**Published:** 2025-01-24

**Authors:** Hao-En Huang, Ombretta Colasanti, Teng-Feng Li, Volker Lohmann

**Affiliations:** 1 Department of Infectious Diseases, Molecular Virology, Section Virus-Host Interactions, Heidelberg University, Medical Faculty Heidelberg 152528 , Heidelberg, Germany; The Ohio State University, Columbus, Ohio, USA

**Keywords:** HAV, NEMO, IKKgamma, innate immunity, hepatocytes, dsRNA, type-I IFN, NF-κB

## Abstract

NF-κB essential modulator (NEMO) is critically involved in the induction of interferons (IFNs) and pro-inflammatory cytokines. Hepatitis A virus (HAV) 3C protease was recently identified to cleave NEMO in non-hepatic cells. This study aimed at understanding efficiency and function of HAV 3C-mediated NEMO cleavage in hepatocytes. HAV 3C protease and its precursor 3CD strongly affected NEMO abundance in ectopic expression models, which was not observed in HAV replicon cells and upon HAV infection. Using a cleavage-resistant NEMO mutant, we found that specific cleavage by 3C only marginally contributed to NEMO degradation, whereas the magnitude of the effect was due to cytotoxic effects induced by 3C activity. Cleavage efficiency generally did not suffice to disrupt the type I IFN or NF-κB signaling pathways. Knockout of NEMO indeed abrogated both pathways, whereas efficient knockdown had limited the impact on NEMO-mediated signaling, suggesting that low levels of NEMO are sufficient to maintain antiviral responses in hepatocytes. NEMO cleavage was barely detectable in a cell line harboring a persistent HAV replicon or in HAV-infected cells. HAV infection induced a robust innate immune response, which was not affected by efficient knockdown of NEMO, arguing for a limited potential contribution of NEMO cleavage to innate immune counteraction. Overall, our data suggest that HAV 3C is capable of partially cleaving NEMO as reported. However, since minute expression levels of NEMO were sufficient for induction of innate immunity, inefficient NEMO cleavage by HAV is unlikely to contribute to dampening of innate immune responses in hepatocytes.

**IMPORTANCE:**

Hepatitis A virus (HAV) establishes acute infections of the liver, which are always cleared, while a number of mechanisms have been identified contributing to immune escape. Among those, proteolytic cleavage of NF-κB essential modulator (NEMO) by HAV has been suggested to counteract innate immune responses. This study demonstrates that the HAV 3C protease cleaves NEMO inefficiently and does not result in substantial disruption of antiviral signaling. Importantly, NEMO remains capable of inducing an effective immune response in hepatocytes even at low expression levels. Our findings suggest a limited role for NEMO cleavage in HAV’s interaction with host immunity and call for a revision of our understanding of HAV counteraction mechanisms.

## INTRODUCTION

Hepatitis A virus (HAV) is a positive-sense single-stranded RNA [(+)ssRNA] virus within the *Picornaviridae* family. Unlike other hepatitis viruses, such as HBV or HCV, that mainly progress to chronic hepatitis, HAV infection leads to a transient inflammation in the liver, which is always cleared within months. Still, fulminant liver failure occurs in a small number of cases, making it a potentially life-threatening disease (1). Open questions remain about how full immune resolution of HAV is established despite inducing only a mild activation of innate immunity (2).

During infection, the (+)ssRNA genome of HAV, acting as a mRNA, is translated into a large polyprotein with a single open reading frame (ORF) and further processed into individual proteins by the viral protease 3C. As other RNA viruses, a negative strand RNA is synthesized during its replication, forming a transient double-stranded RNA (dsRNA) intermediate (3). DsRNA can be detected by several pattern recognition receptors (PRRs): toll-like-receptor 3 (TLR3) in endosomes and RIG-I-like receptors (RLRs) in the cytoplasm, which include retinoic acid-inducible gene I (RIG-I), melanoma differentiation-associated protein 5 (MDA5), and laboratory of genetics and physiology 2 (LGP2), leading to the establishment of an antiviral state. This gives rise to the activation of interferon (IFN) regulatory factors (IRFs), in particular IRF3, resulting in the induction of IFN-stimulated genes (ISGs) and the secretion of type I IFNs (4). Importantly, nuclear factor-κB (NF-κB) is also activated downstream of the PRR activation, promoting the expression of inflammatory chemokines. NF-κB is a versatile transcription factor that plays a crucial role in regulating inflammation, immune responses, cell survival, and proliferation in various cell types (5), which can also be activated by the tumor necrosis factor receptor (TNFR) (6). NF-κB activation involves the IκB kinase (IKK) complex, which facilitates the degradation of the inhibitor IκBα, allowing NF-κB to translocate to the nucleus and initiate the transcription of inflammatory cytokine genes (7). NF-κB essential modulator (NEMO, IKKgamma), a noncatalytic 48 kDa protein, is one of the components of the IKK complex, which acts as a regulator of the IKK complex and its upstream activators (8). In addition to modulating the NF-κB signaling, NEMO was also demonstrated to contribute specifically to the RLR pathway where it interacts with the adaptor mitochondrial antiviral-signaling protein (MAVS), activating IRFs and the IFN signaling pathway (9). Therefore, NEMO appears as a pivotal factor, mediating innate immune responses upon viral infection. Despite its importance, NEMO was shown to participate in both the NF-κB and IRF signaling pathways in response to RNA viral infection exclusively in HEK293T cells and mouse embryonic fibroblasts (MEFs) (9). Investigation of NEMO in hepatocytes was so far focused on its involvement in liver injury (10), hepatocellular carcinoma (HCC) (11, 12) and HBV infection (13).

Previously, it has been suggested that HAV developed several strategies to counteract innate immunity by protease cleavage of proteins involved in signaling, including the important adaptor molecules MAVS, TIR domain-containing adapter inducing IFN-β (TRIF), and NEMO (14–16). NEMO cleavage by HAV 3C protease at position Q304 was shown to disrupt the RLR signaling pathways in HEK293T cells (16). However, the functional relevance of these cleavage events has been challenged by our recent study, demonstrating that HAV is efficiently sensed by MDA5 and LGP2, driving downstream ISG expression with inefficient counteraction of MAVS due to limited proteolytic cleavage (17). Therefore, it remains controversial to which extent HAV functionally counteracts the interferon response.

Here, we aimed at characterizing HAV 3C protease cleavage efficiency of NEMO and its functional impact on innate immunity and viral replication in human liver-derived cell culture systems. We showed that ectopic overexpression of 3C resulted in only partial NEMO cleavage, which did not significantly affect the type-I IFN signaling pathway and had only a mild impact on the NF-κB pathway. In addition, we demonstrate that NEMO could still modulate and promote innate immunity even at very low expression levels. Altogether, our data suggest that the 3C protease cleavage of NEMO might not be a major mechanism for HAV to evade innate immunity, which alters our current understanding of the counteraction mechanisms during HAV infection.

## RESULTS

### Overexpression of NEMO suppresses innate immune response in Huh7.5 cells

We recently showed the HAV induces a decent cell intrinsic innate immune response in hepatocytes via MDA5/LGP2, with limited impact of proteolytic cleavage of TRIF and MAVS (17). Wang et al. previously demonstrated that NEMO is cleaved by the 3C protease when overexpressed in HEK293T cells (16). Due to the robust ISG induction mounted upon HAV infection, we aimed to revisit NEMO cleavage by 3C and its impact on the host’s innate immune and inflammatory responses in more authentic liver-derived models, including HCC-derived Huh7 and HepG2 cells, both permissive for HAV infection, and immortalized, non-neoplastic PH5CH hepatocytes.

The Huh7 subclone Huh7.5 lacks functional expression of PRRs (18–20) and therefore allowed the analysis of NEMO functions in different signaling pathways separately. To this end, we used Huh7.5 cell lines reconstituted with TLR3, RIG-I, or MDA5 expression (17), respectively, stably expressing NEMO or HA-tagged NEMO under transcriptional control of the EF1α promoter.

We first aimed to determine the potential functional impact of increased NEMO abundance, since it has been shown that overexpression (OE) of NEMO could hinder NF-κB signaling (21), while in other models a wide range of NEMO expression was compatible with signaling (22). Three genes were analyzed to monitor NEMO function upon stimulation with the synthetic dsRNA polyinosinic–polycytidylic acid (poly(I:C)): *IFIT1* as a correlate of IRF3 induction (23), the pro-inflammatory chemokine *CXCL10*, downstream of both NF-κB (24) and IRF3 activation (25) and *TNFAIP3*, specifically downstream of the NF-κB pathway (26).

**Fig 1:**
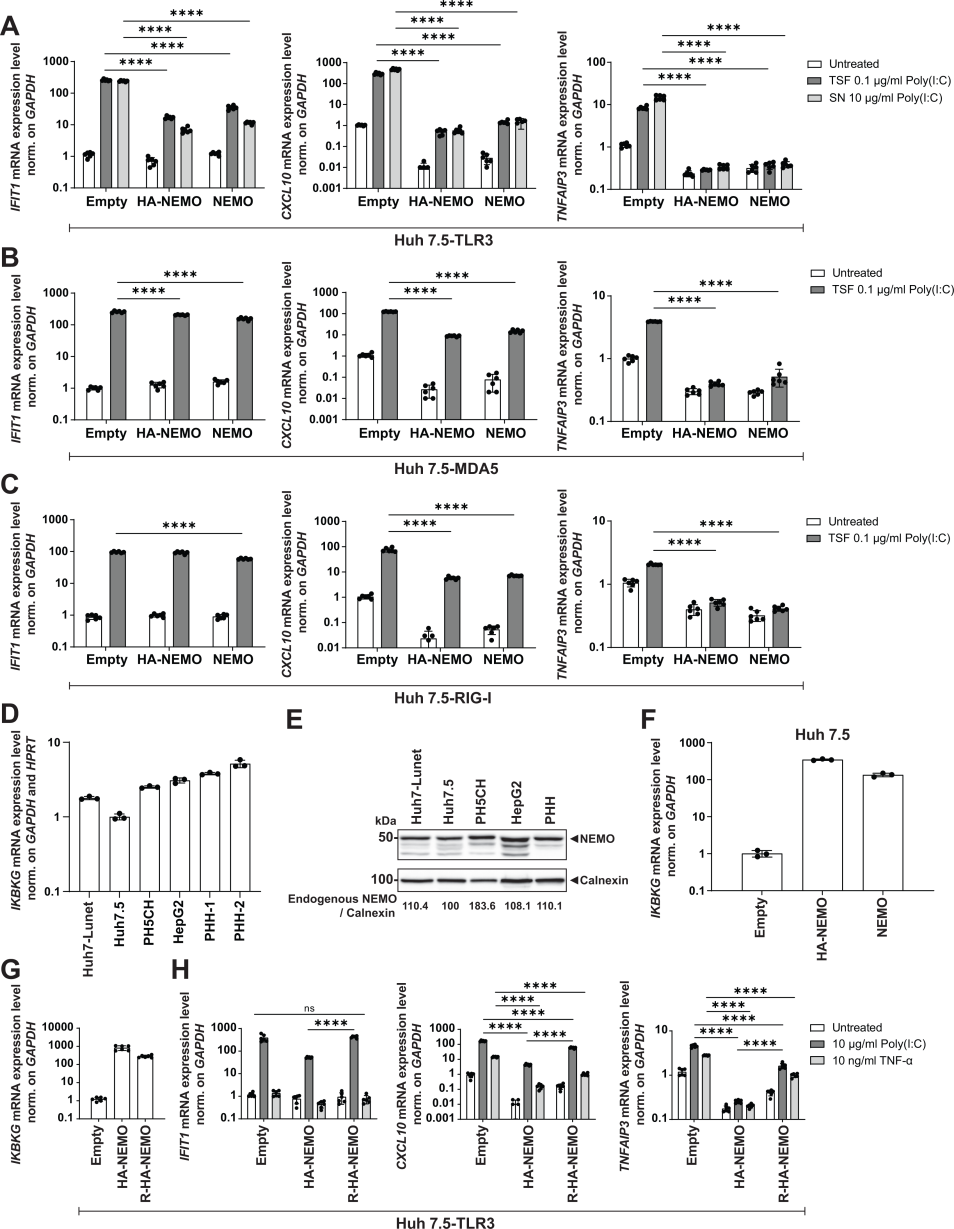
Establishment of a NEMO OE system. (A–C) NEMO- or HA-NEMO-overexpressing stable Huh7.5 cells reconstituted with the respective PRR were stimulated with 0.1 µg/mL poly(I:C) by transfection (TSF), 10 µg/mL poly(I:C) in the supernatant (SN), or the transfection reagent (untreated) for 6 h. Total RNA was isolated, and mRNA levels of the respective gene were quantified by RT-qPCR. Values were normalized to the untreated empty control to demonstrate the downregulating effect of NEMO OE. (**D**) *IKBKG* mRNA levels of four different liver-derived cell lines and PHH were measured by RT-qPCR and normalized on two housekeeping genes, *GAPDH* and *HPRT* (27). (**E**) Four different liver-derived cell and PHH cell lysates were subjected to Western blot analysis with anti-NEMO and anti-Calnexin antibodies. Calnexin was used as a loading control. The band intensities were quantified using Fiji and normalized to the respective Calnexin bands. The ratios indicated below blots were relative to the expression level in Huh7.5 (set as 100). (**F**) Huh7.5 cells were transduced with lentiviral vectors encoding only the selectable marker (empty), NEMO, or HA-NEMO. *IKBKG* mRNA levels were measured by RT-qPCR. (**G and H**) Selected Huh7.5–TLR3 cells were transiently transduced with lentiviral vectors encoding either the selection marker (empty) or HA-NEMO under transcriptional control of either the EF1α promoter (HA-NEMO) or ROSA26 promoter (R-HA-NEMO). After stimulation of 10 µg/mL poly(I:C) or 10 ng/mL TNF-α in the SN for 6 h, total RNA was isolated, and mRNA levels of *IKBKG* (**G**), *IFIT1*, *CXCL10*, and *TNFAIP3* (**H**) were quantified by RT-qPCR. *GAPDH* was used as a reference gene. Values were normalized to the empty control. Data represent mean ± SD of biological duplicates with technical triplicates (A–C, G–H) or of technical triplicates (**D and F**). Statistical analysis was performed with multiple *t*-test. ****, *P* < 0.0001. ns (non-significant); OE (overexpression).

Indeed, induction of all marker genes was significantly affected when NEMO or HA-NEMO were overexpressed, albeit to varying extent (Fig. 1A through C). While *TNFAIP3* and *CXCL10* induction were abrogated or reduced by more than 10-fold, respectively, in case of all PRRs (Fig. 1A through C, middle and right panels), *IFIT1* induction was strongly impaired only upon TLR3 activation (Fig. 1A through C, left panels).

Altogether, we found that NEMO OE completely inhibited the NF-κB and in part the IRF3 pathway, particularly in TLR3-reconstituted cells. As both NEMO and HA-NEMO-expressing cell lines responded to poly(I:C) at a similar level, the HA-tag appeared not to interfere with NEMO functions.

Next, we aimed to assess physiological NEMO expression levels (encoded by *IKBKG*) in various hepatocytes, by comparing mRNA and protein expression in two Huh7 subclones (Huh7.5 and Huh7-Lunet) as well as in PH5CH, HepG2 and primary human hepatocytes (PHH). Overall, *IKBKG* mRNA levels were lowest in Huh7.5 cells, but overall similar, within a fivefold range (Fig. 1D). NEMO protein expression was even more homogenous, within a twofold range, being highest in non-neoplastic immortalized PH5CH cells (Fig. 1E). However, OE of NEMO or HA-NEMO by lentiviral transduction resulted in ca. 100-fold increased mRNA levels compared with naïve Huh7.5 cells (Fig. 1F). In order to rescue the inhibitory effect given by OE of NEMO, we tested a weaker promoter, ROSA26 (28), in the context of Huh7.5–TLR3 cells, where NEMO OE had the strongest impact. The ROSA-HA-NEMO (R-HA-NEMO)-expressing cells showed roughly 10-fold less *IKBKG* expression compared with EF1α-regulated HA-NEMO (Fig. 1G). Upon poly(I:C) stimulation, R-HA-NEMO had no impact on *IFIT1* induction and significantly rescued *CXCL10* and *TNFAIP3* induction, albeit still reduced their expression (Fig. 1H). TNF-α treatment did not induce *IFIT1*, as expected, and moderately upregulated expression of *CXCL10* and *TNFAIP3*, which was affected by R-HA-NEMO expression, resulting in a very small window (Fig. 1H).

These data indicated that Huh7.5–TLR3 cells expressing R-HA-NEMO were an appropriate tool for studying 3C protease counteraction to the function of NEMO upon poly(I:C) stimulation.

### HAV 3C protease lacks counteraction to ISG expression

**Fig 2:**
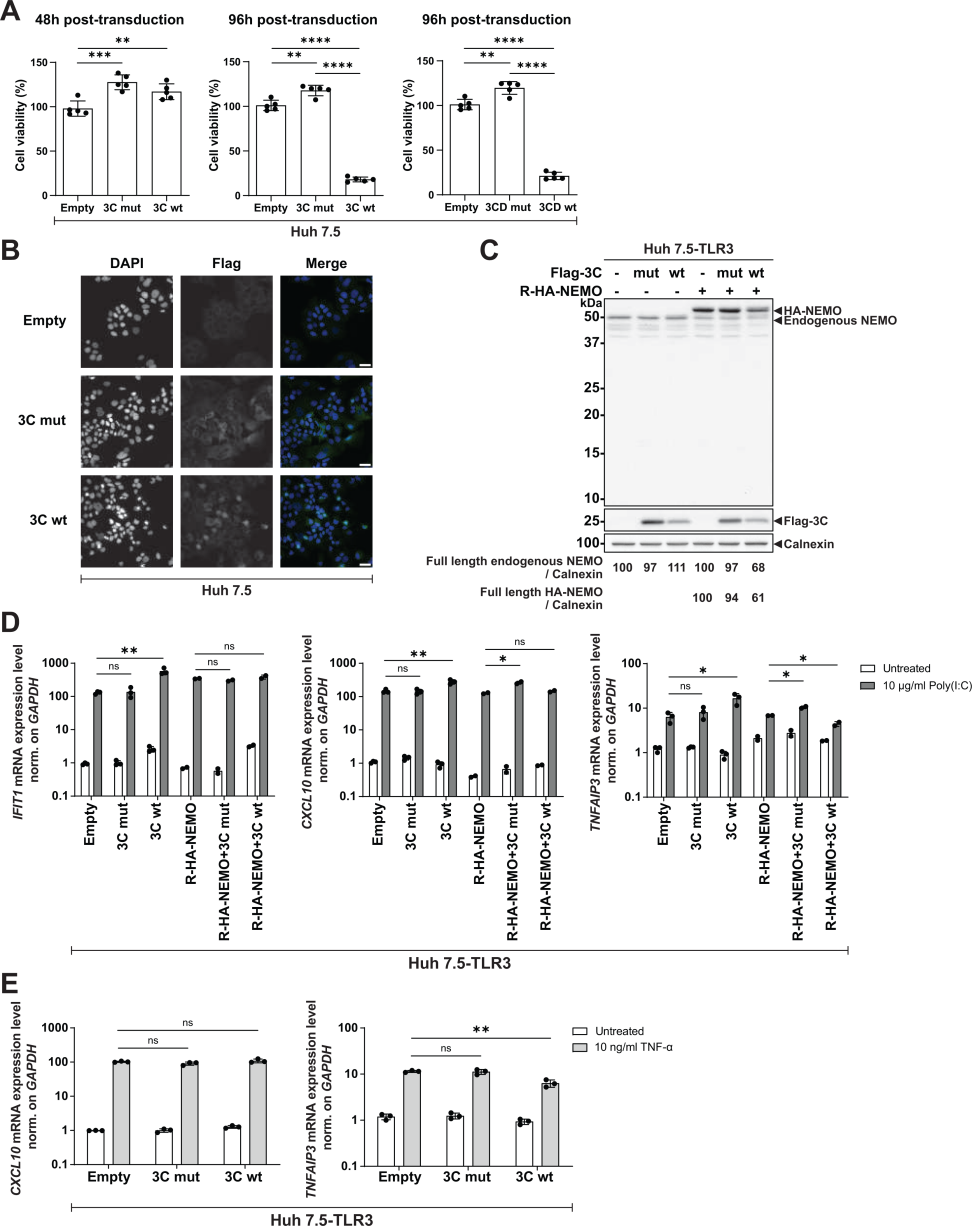
Functional analysis of HAV 3C counteraction of NEMO in Huh7.5–TLR3 cells. (**A**) Huh7.5 cells were transduced with lentiviral vectors encoding a selection marker (empty), Flag-3C mut/wt, or Flag-3CD mut/wt protease. After 48 h (left panel) or 96 h (middle and right panels), cells were subjected to the analysis of viable cells using the CellTiter-Glo assay. Values were normalized to the empty control (set as 100%). Data represent mean ± SD of five biological replicates. (**B**) Cells were transduced as in (**A**) for 48 h. Cells were stained with anti-Flag (green). Nuclei were stained with DAPI. Images were taken at 40×. Scale bar, 40 µm. (**C**) Huh7.5–TLR3 cells were transiently transduced with lentiviral vectors encoding R-HA-NEMO and Flag-3C mut or Flag-3C wt. Cell lysates were subjected to Western blot analysis using anti-NEMO, anti-Flag, and anti-Calnexin antibodies. Calnexin was used as a loading control. The band intensities were quantified using Fiji and normalized to the respective Calnexin bands. The ratios indicated below blots were relative to the mock control (set as 100). Data are representative experiments from *n* = 2 biological replicates. (**D and E**) Huh7.5–TLR3 cells were transduced as in (**C**). After stimulation with 10 µg/mL poly(I:C) (**D**), 10 ng/mL TNF-α (**E**) in the supernatant, or left untreated for 6 h, total RNA was isolated, and the respective mRNA levels were quantified by RT-qPCR. GAPDH was used as a reference gene. Values were normalized to the empty control. Data represent mean ± SD of biological duplicates or triplicates with technical triplicates. Statistical analysis was performed with Welch’s *t*-test (**A**) or with multiple *t*-test (**D and E**). *, *P* < 0.05; **, *P* < 0.01; ***, *P* < 0.001; ****, *P* < 0.0001. mut (mutant); wt (wildtype); ns (non-significant).

Next, we sought to express the HAV 3C protease through lentiviral transduction in Huh7.5 cells to study its functional impact on the host signaling pathways. Given that HAV 3C was reported to be cytotoxic (29, 30), we first measured the cell viability at different timepoints post-transduction. Consistently, we observed low cell viability upon 3C and 3CD wild-type (wt) expression at 96 h post-transduction but not for the catalytically inactive 3C mutant C172A (31), while no cytotoxicity was detectable at 48 h (Fig. 2A). We performed immunofluorescence (IF) analysis to confirm detectable protease expression at 48 h after transduction (Fig. 2B) and chose this timepoint for further experiments. Still, the cytotoxicity induced by 3Cwt and 3CDwt explains the overall lower expression levels compared with the mutant proteins observed throughout all experiments based on ectopic overexpression.

To study NEMO cleavage and the functional impact on the TLR3 pathway, we expressed the wt and mutant 3C protease, either with or without R-HA-NEMO in Huh7.5–TLR3 cells. Wt 3C affected the abundance of full-length endogenous NEMO and the ectopically expressed variant (Fig. 2C). However, we detected no visible cleavage products in both scenarios. *IFIT1*, *CXCL10*, and *TNFAIP3* expression was not significantly reduced upon poly(I:C) stimulation by 3C wt compared with 3C mutant (Fig. 2D), suggesting an inefficient counteraction of the TLR3 pathway by 3C wt protease. We further checked the integrity of the NF-κB pathway upon TNF-α stimulation in absence of ectopic NEMO expression and observed no downregulation of *CXCL10* but a slight reduction of *TNFAIP3* (around 1.8-fold) upon 3C wt OE (Fig. 2E).

In summary, transient expression of 3C protease by lentiviral transduction did not result in detectable NEMO cleavage and had little impact on TLR3 activation.

### NEMO abundance is reduced by HAV 3C protease activity in a transient overexpression system

To overcome the problems associated with the toxicity of 3C, we next chose an OE system in Huh7-Lunet cells stably expressing the T7 RNA polymerase. This model provides a high expression level of the delivered genes and allows analysis of the functions of the viral proteases with peak expression already at 24 h after transfection (17).

**Fig 3:**
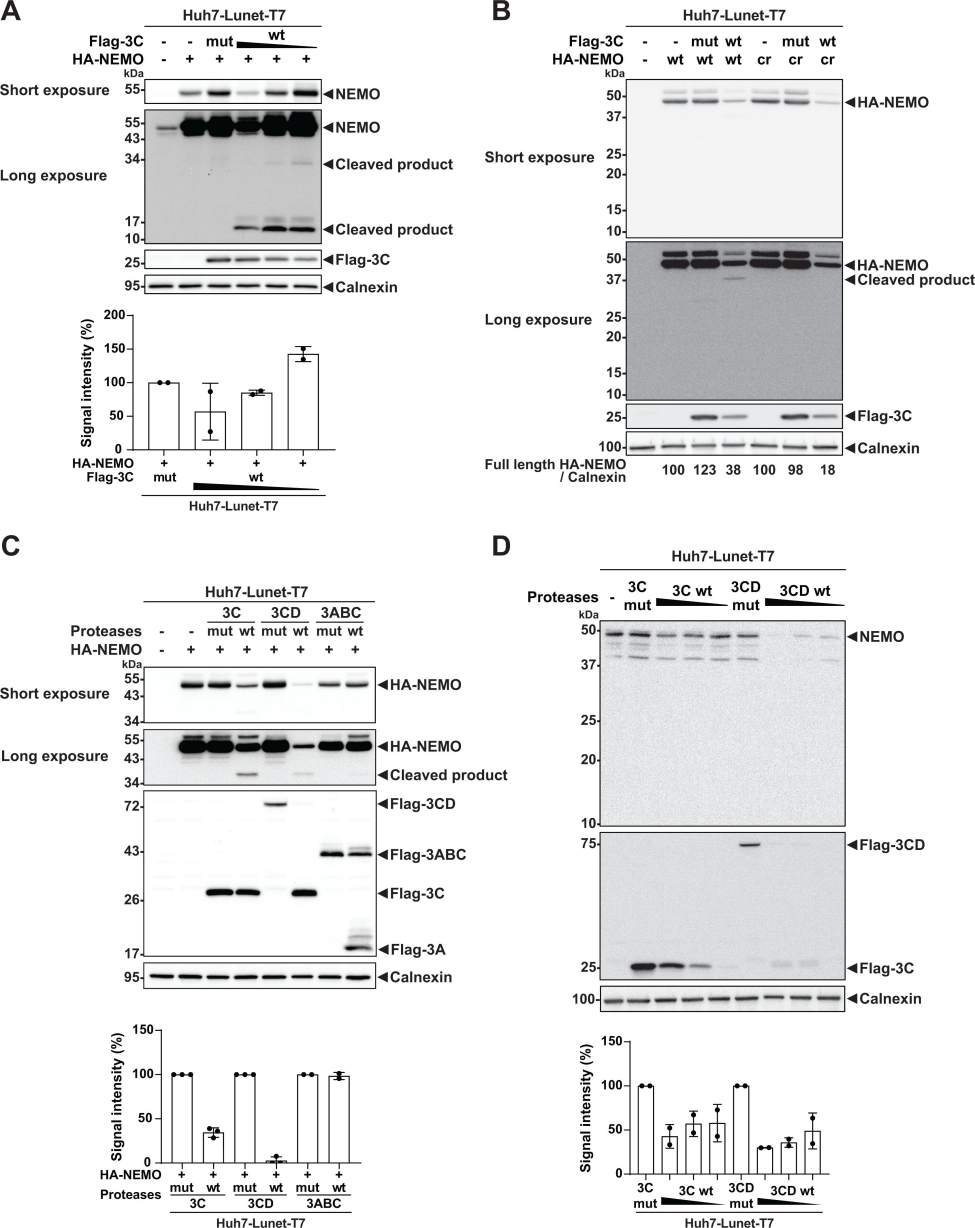
Detection of NEMO cleavage by HAV proteases. (**A**) Huh7-Lunet cells stably expressing T7 polymerase (Huh7-Lunet-T7) were co-transfected with individual pTM plasmids encoding HA-NEMO (1 µg) along with either Flag-3C mut (1.5 µg) or Flag-3C wt (1.5, 1, and 0.5 µg) for 24 h. Cell lysates were subjected to Western blot analysis using anti-NEMO, anti-Flag, and anti-Calnexin antibodies. (**B**) Huh7-Lunet-T7 cells were co-transfected with individual pTM plasmids encoding HA-NEMO wt or HA-NEMO cr (1 µg) along with either Flag-3C mut or Flag-3C wt (1.5 µg) for 24 h. Cell lysates were subjected to Western blot analysis using anti-HA, anti-Flag, and anti-Calnexin antibodies. (**C**) Huh7-Lunet-T7 cells were co-transfected with pTM plasmids encoding HA-NEMO (1 µg) and Flag-tagged proteases (1.5 µg) for 24 h. Cell lysates were subjected to Western blot analysis using anti-HA, anti-Flag, and anti-Calnexin antibodies. (**D**) Huh7-Lunet-T7 cells were transfected with pTM plasmids encoding Flag-3C mut, Flag-3C wt (1.5 µg), Flag-3CD mut, and Flag-3CD wt (1.5, 1, and 0.5 µg) for 24 h. Cell lysates were subjected to Western blot analysis using anti-NEMO, anti-Flag, and anti-Calnexin antibodies. Calnexin was used as a loading control. Data show a representative experiment from *n* = 2 or 3 biological replicates. The bottom panels of the figures indicate the quantification of full-length NEMO band intensities from all replicates. The band intensities were quantified using Fiji and normalized to the respective Calnexin bands, and the ratios are shown relative to the respective mut band intensity (set as 100). Data represent mean ± SD of *n* = 2 or 3 biological replicates. mut (mutant); wt (wildtype); cr (cleavage-resistant).

To detect NEMO cleavage, we co-transfected increasing amounts of a plasmid encoding a Flag-tagged 3C protease with fixed amounts of a plasmid encoding HA-tagged NEMO, both under the control of the T7 promoter, into Huh7-Lunet-T7 cells (17). We chose this co-transfection approach, assuming that transfected cells would obtain both plasmids, since the amount of endogenous NEMO cleavage was formally limited by transfection efficiency, which was estimated approximately 50% based on 3C expression (Fig. S1A). We observed that NEMO was partially cleaved to a maximum of 50% by wt 3C, but not by the 3C mutant, with cleavage products being detectable at around 35 and 15 kDa upon longer exposure of the blot (Fig. 3A). However, quantification of NEMO cleavage in this setting appeared challenging, since the abundance of cleavage products declined to a similar extent as full-length NEMO with ascending expression of 3C wt.

The lack of correlation between NEMO full-length protein abundance and cleavage products prompted us to assess the specificity of NEMO cleavage by 3C. To this end, we generated a previously described NEMO mutant, Q304A (16), which was resistant to specific 3C-mediated NEMO cleavage (NEMO cr) (16). Interestingly, full-length NEMO was diminished to a similar extend for HA-NEMO wt and cr, but in the absence of any detectable cleavage product in case of the HA-NEMO cr (Fig. 3B). Still, reduced amounts of full-length HA-NEMO were only found with 3C wt, indicating that NEMO degradation was linked to 3C protease activity. This result suggested that the majority of reduced NEMO abundance was not due to specific proteolytic cleavage by 3C but rather mediated by cellular processes initiated by the 3C protease.

Previously, NEMO was also shown to be cleaved by the 3C protease precursors 3CD and 3ABC since both harbor proteolytic activity, albeit both precursors were slightly less efficient (16). NEMO abundance was indeed strongly reduced by 3C and 3CD, but not by 3ABC. 3CD was even more efficient than 3C, reducing amounts of ectopically expressed NEMO by more than 90%, albeit also here, almost no cleavage products were detectable (Fig. 3C). Overall, the pattern of reduced NEMO abundance matched very well to the observed cytotoxicity of 3C wt/3CD wt expression and the lack of cytotoxicity found for 3ABC in a previous study (17), suggesting that unspecific degradation of NEMO was indeed induced by cytotoxic effects associated with expression of active 3C/3CD. We next aimed to quantify endogenous NEMO cleavage upon protease OE. The abundance of endogenous NEMO was reduced dose-dependently by expression of wt 3C and 3CD protease to about 50% (Fig. 3D), likely limited by transfection efficiency (Fig. S1A). Finally, we assessed whether the reduced abundance of endogenous NEMO upon 3C or 3CD expression impaired TLR3 signaling. However, despite the observed substantially diminished amounts of NEMO (Fig. 3D), *IFIT1* and *CXCL10* induction were only marginally affected compared with 3C/3CD mutant, or empty vector, whereas *TNFAIP3* induction was slightly but significantly reduced (Fig. S1B).

Taken together, we observed that the abundance of NEMO was strongly reduced by the HAV 3C protease and more prominently by its precursor 3CD, but not by 3ABC, in an OE model based on Huh7 cells. Due to low amounts of cleavage products observed for NEMO wt and the same reduction observed for a 3C cleavage resistant NEMO variant, it seems likely that the majority of NEMO degradation was caused by cellular protease activities induced by the cytotoxic effects of 3C and 3CD expression in this model.

### Incomplete cleavage of NEMO and induction of ISGs in HAV-positive cells

Since we observed limited specific NEMO cleavage, but a substantial impact on NEMO abundance by OE of 3C protease and the precursor 3CD in a transfection-based OE model (Fig. 3C and D), we next aimed to study NEMO cleavage and its impact on innate immunity in more physiological models, including HAV replication.

**Fig 4:**
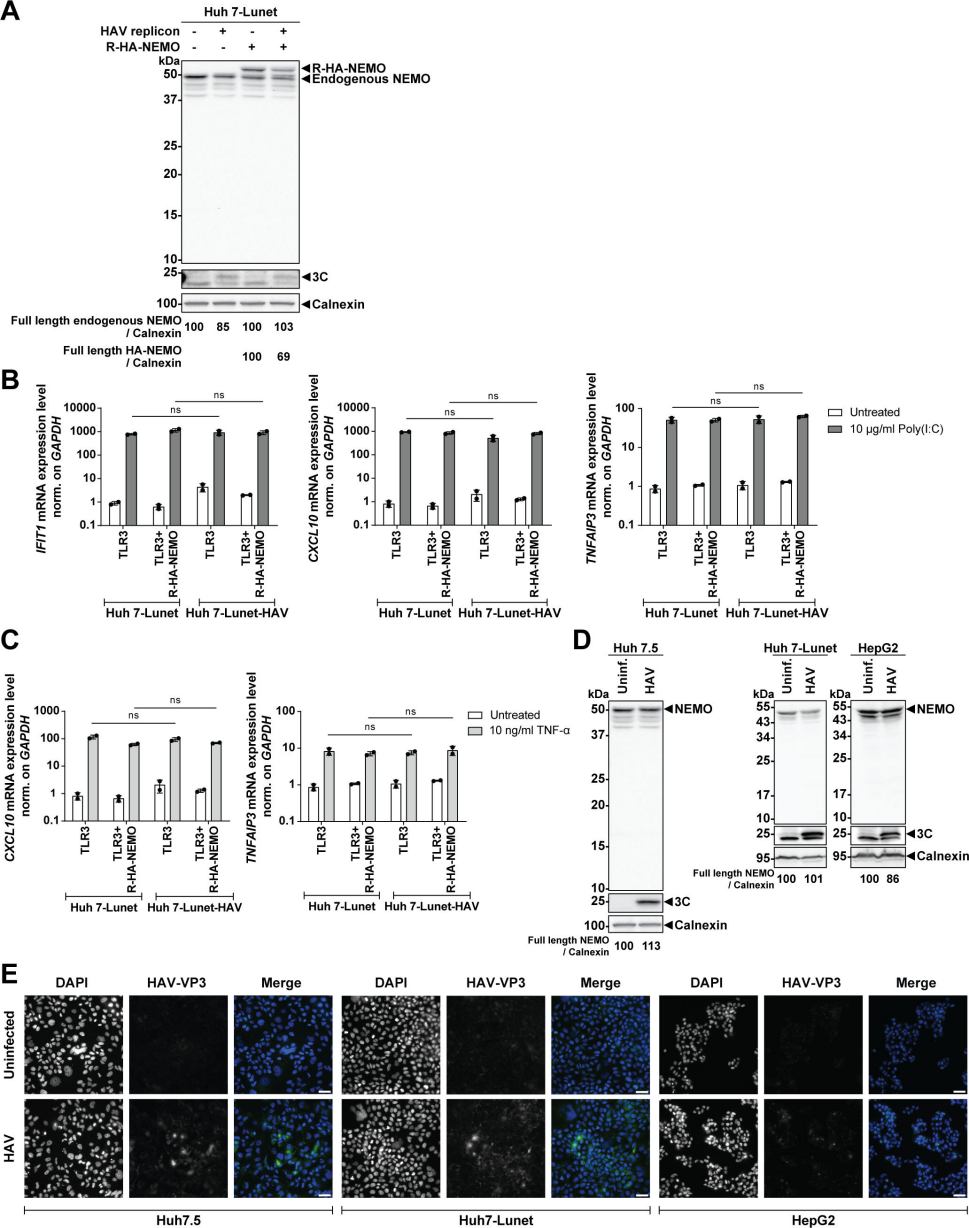
Analysis of HAV 3C counteraction of NEMO in HAV-positive cells. (**A**) Huh7-Lunet naïve cells and cells expressing HAV replicons were transiently transduced with the lentiviral vectors encoding TLR3 and R-HA-NEMO. Cell lysates were subjected to Western blot analysis with anti-NEMO, anti-3C, and anti-Calnexin antibodies. (**B and C**) Cells were transduced as described in (**A**). After stimulation with 10 µg/mL poly(I:C) (**B**), 10 ng/mL TNF-α (**C**) in the supernatant, or left untreated for 6 h, total RNA was isolated, and the respective mRNA levels were quantified by RT-qPCR. *GAPDH* was used as a reference gene. Values were normalized to the mock control. Data represent mean ± SD of biological duplicates with technical triplicates. Statistical analysis was performed with multiple *t*-test. (**D**) The hepatic cell lines Huh7.5, Huh7-Lunet (MOI = 1.4), and HepG2 (MOI = 0.4) were infected with HAV for 96 h, harvested for Western blot analysis with anti-NEMO, anti-3C, and anti-Calnexin antibodies. (**E**) The indicated cell lines were infected with HAV as in (**D**). Cells were stained with anti-HAV-VP3 (green). Nuclei were stained with DAPI. Images were taken at 40×. Scale bar, 40 µm. (**A and D**) Calnexin was used as a loading control. The band intensities were quantified using Fiji and normalized to the respective Calnexin bands. The ratios indicated below blots were relative to the respective mock control (set as 100). Data are representative experiments from *n* = 1 or 2 biological replicates. ns (non-significant).

We first chose previously established Huh7-Lunet cells harboring a selectable, persistent HAV replicon (32), where, due to the selection process, all cells express 3C protease to assess NEMO cleavage. A minor reduction of endogenous and ectopically expressed NEMO protein levels and no cleavage products were observed in replicon cells compared with naïve Huh7-Lunet, with detectable expression of 3C (Fig. 4A). Slight increases in the amounts of endogenous NEMO might be due a ca. twofold increase of IKBKG mRNA abundance in HAV replicon cells or in cell expression 3C protease (Fig. S2), which might also compensate to some extent loss of NEMO protein by the 3C protease. Next, we reconstituted TLR3 expression and overexpressed R-HA-NEMO transiently by lentivirus transduction and stimulated the cells with poly(I:C) and TNF-α, aiming at measuring a potential impact of HAV on the IRF3 and NF-κB pathways. The HAV-replicon cells were still able to mount a full response to poly(I:C) and TNF-α for *IFIT1*, *CXCL10*, and *TNFAIP3*, regardless of the expression of 3C and its precursors in all cells, indicating a general lack of strong innate immune counteraction given by HAV non-structural proteins, in line with literature (17) (Fig. 4B and C).

We next infected different liver-derived cell lines, Huh7.5, Huh7-Lunet, and HepG2 cells with HAV and quantified full-length endogenous NEMO protein expression 96 h post-infection. No reduction in NEMO abundance was found in Huh7.5 and Huh7-Lunet cells, and only a slight decrease (14%) in HepG2 cells (Fig. 4D), although at this time point, typically 30%–40% of cells contained detectable antigen levels (Fig. 4E).

In summary, we demonstrated that NEMO amounts were not substantially affected by HAV 3C protease and its precursors in authentic HAV replication models.

### Low expression level of NEMO suffices to induce ISGs in hepatocytes

**Fig 5:**
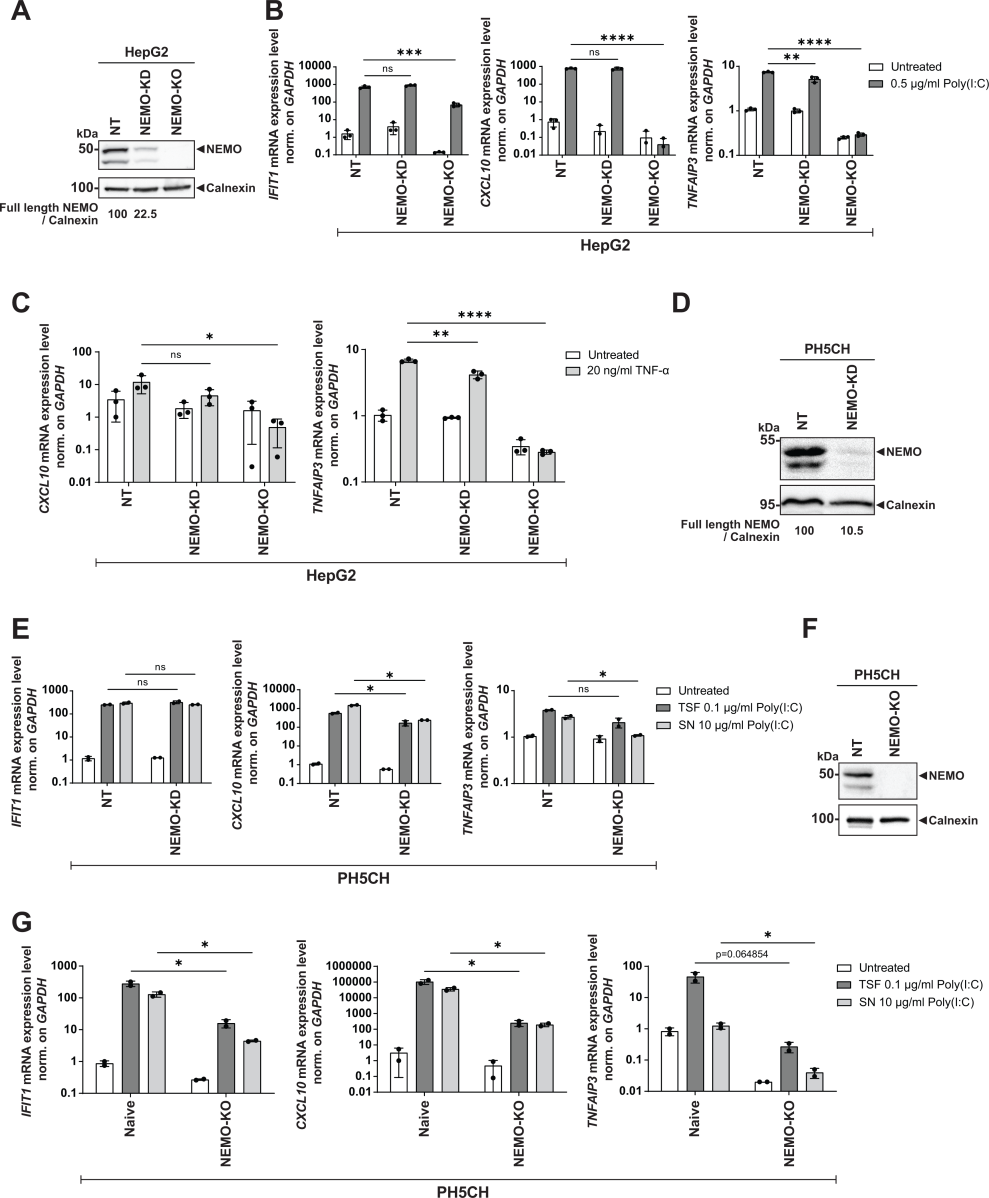
Characterization of innate immunity upon KD and KO of NEMO in liver-derived cell lines. (**A**) HepG2 cells were transduced with lentiviral vectors encoding non-targeting (NT) or *IKBKG* sgRNA (NEMO-KO) sequences and selected for single-cell clones. The selected NT cell clone was transfected with either a non-targeting siRNA (NT) or the *IKBKG* siRNA (NEMO-KD) for 48 h. The cells were lysed and collected for Western blot analysis with anti-NEMO and anti-Calnexin antibodies. (**B and C**) HepG2 cells described in (**A**) were stimulated with either 0.5 µg/mL poly(I:C) by transfection (**B**), 20 ng/mL TNF-α in the supernatant for 3 h (**C**), or left untreated. Total RNA was isolated, and the respective mRNA levels were quantified by RT-qPCR. Note that *IFIT1* was not analyzed due to the absence of induction by TNF-α (see Fig. 1H). (**D**) NT and NEMO-KD PH5CH cells were generated as described in (**A**). The cells were harvested for Western blot analysis with anti-NEMO and anti-Calnexin antibodies. (**E**) PH5CH cells described in (**D**) were stimulated with 0.1 µg/mL poly(I:C) by transfection (TSF), with 10 µg/mL poly(I:C) in supernatant (SN), or the transfection reagent (untreated) for 6 h. Total RNA was isolated, and the respective mRNA levels were quantified by RT-qPCR. (**F**) NT and NEMO-KO PH5CH clone were generated as described in (**A**). The cells were harvested for Western blot analysis with anti-NEMO and anti-Calnexin antibodies. (**G**) PH5CH naïve cells and NEMO-KO clone were stimulated with poly(I:C) as in (**E**). Total RNA was isolated, and the respective gene levels were quantified by RT-qPCR. GAPDH was used as a reference gene. Values were normalized to the naïve cell control. Data represent mean ± SD of biological duplicates with technical triplicates. (**A, D, F**) Calnexin was used as a loading control. The band intensities were quantified using Fiji and normalized to the respective Calnexin bands. The ratios indicated below blots were relative to the NT control (set as 100). Statistical analysis was performed with multiple *t*-test. *, *P* < 0.05; **, *P* < 0.01; ***, *P* < 0.001; ****, *P* < 0.0001. ns (non-significant).

To elucidate the role of NEMO in innate immune induction in liver-based cell culture models, we knocked down (KD) and knocked out (KO) NEMO in the hepatoma cell line HepG2 (Fig. 5A) (33). KD reduced NEMO expression by approximately 80% but surprisingly did not affect *IFIT1* and *CXCL10* induction and only marginally reduced expression of the NF-κB-specific gene *TNFAIP3* upon poly(I:C) transfection (Fig. 5B). In contrast, NEMO-KO resulted in a complete abrogation of *CXCL10* and *TNFAIP3* expression and a strong reduction of *IFIT1* mRNA expression after induction (Fig. 5B). Similarly, the knockdown of NEMO slightly affected TNF-α treatment, while the knockout substantially decreased the expression of *CXCL10* and *TNFAIP3*, although *CXCL10* induction was very low in HepG2 cells for unknown reasons (Fig. 5C).

Next, we assessed the function of NEMO in PH5CH cells, an immunocompetent immortalized non-neoplastic liver-derived cell line expressing TLR3 endogenously (34), in contrast to HepG2 cells (17, 35, 36). KD of NEMO in PH5CH cells was efficient (90%, Fig. 5D) and revealed comparable results to HepG2 upon stimulation with poly(I:C). For *IFIT1*, neither way of poly(I:C) stimulation, transfection addressing all PRRs, nor TLR3-specific supernatant feeding, was sensitive to NEMO-KD, suggesting an intact IRF3 signaling (Fig. 5E, left panel). *CXCL10* expression was slightly reduced with both means of poly(I:C) stimulation, whereas, interestingly, *TNFAIP3* showed a significant downregulation only when TLR3 was stimulated (Fig. 5E, middle and right panels), albeit at very low general induction levels. Again, KO of NEMO in the same cells (Fig. 5F) resulted in a dramatic reduction of RLR- and TLR3-induced *IFIT1* and *CXCL10* induction. The overall far higher apparent induction levels observed here compared with NEMO-KD were due to differences in basal *CXCL10* expression in PH5CH naïve compared with the NT reference cell lines used (Fig. 5G and E, middle panels). *TNFAIP3* expression was already reduced without stimulus and slightly induced after poly(I:C) transfection, remaining below the level of naïve cells, but induction was abrogated after supernatant delivery of poly(I:C) (Fig. 5G). The fact that *CXCL10* and *TNFAIP3* induction upon poly(I:C) transfection was completely abrogated in HepG2-NEMO-KO cells (Fig. 5B) but remained slightly inducible in PH5CH-NEMO-KO (Fig. 5G) might be due to differences in IRF3/NF-κB pathway usage and/or alternative induction routes in PH5CH.

Altogether, these data suggested that NEMO could function at a low expression level to maintain the IRF3 and NF-κB signaling in hepatocytes. KO of NEMO partially impaired the IRF3 signaling but completely disrupted the NF-κB pathway, arguing for an important or vital contribution, respectively.

### Low expression level of NEMO is sufficient to drive HAV-induced innate immune response

**Fig 6:**
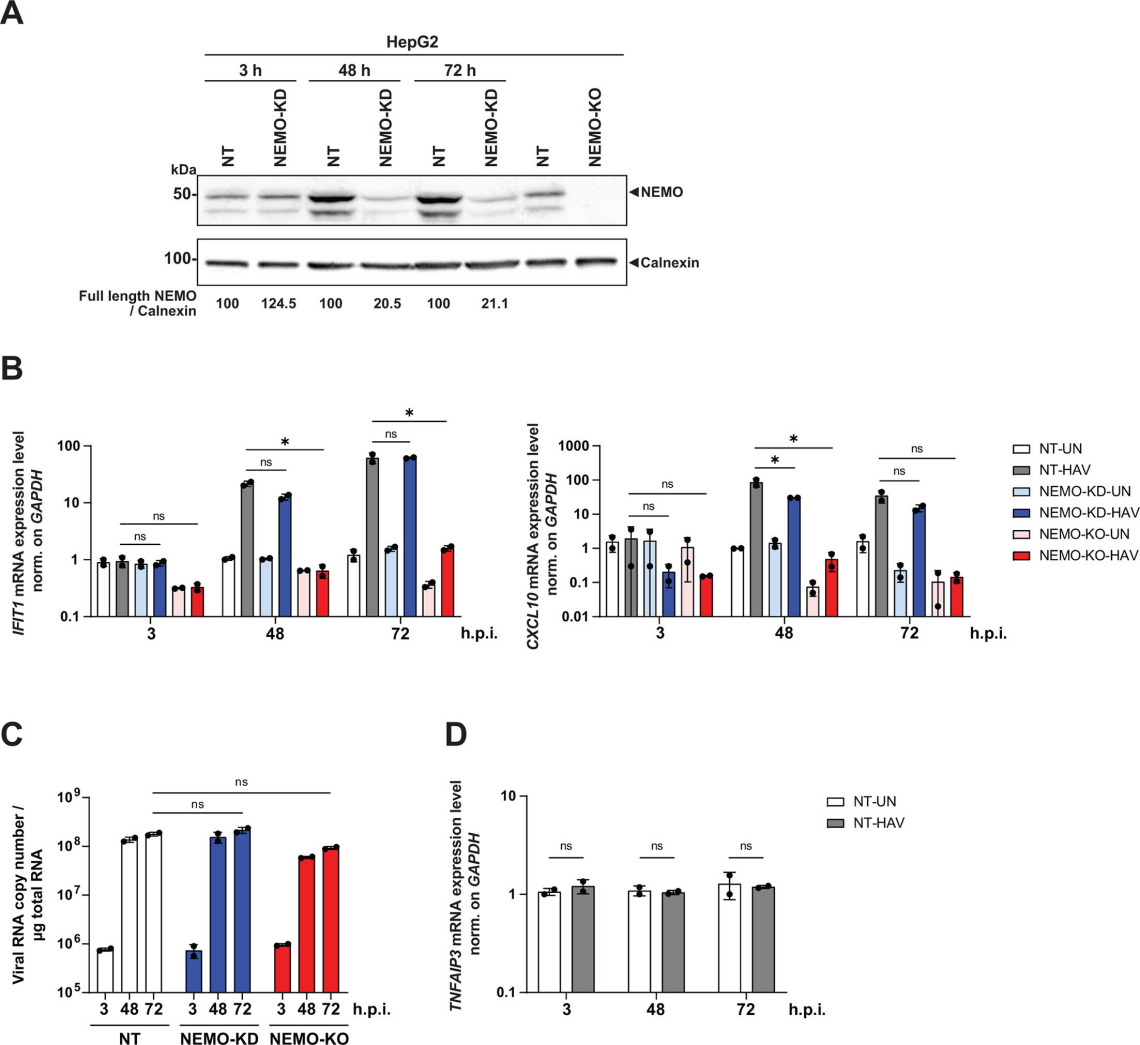
Impact of KD and KO of NEMO on the innate immune response in HAV-infected HepG2 cells. (**A**) HepG2 cells were treated as in Fig. 5A and harvested at the indicated timepoints for Western blot analysis with anti-NEMO and anti-Calnexin antibodies. Calnexin was used as loading control. The band intensities were quantified using Fiji and normalized to the respective Calnexin bands. The ratios indicated below blots were relative to the respective mock control (set as 100). (**B and C**) The selected NT and NEMO-KO HepG2 clones depicted in (**A**) were infected with HAV (MOI = 1) for 1 h, and the NT clone was transfected with non-targeting siRNA or the *IKBKG* siRNA (NEMO-KD). The cells were harvested at the indicated timepoints for total RNA extraction and subject to *IFIT1, CXCL10* mRNA (**B**), and HAV RNA (**C**) quantification by RT-qPCR. (**D**) *TNFAIP3* gene level was measured by RT-qPCR in the NT cells described in (**B**) and (**C**). *GAPDH* was used as a reference gene. Values were normalized to the mock control. Data represent mean ± SD of biological duplicates with technical triplicates. Statistical analysis was performed with multiple *t*-test. *, *P* < 0.05. ns (non-significant); h.p.i. (h post-infection).

**Fig 7:**
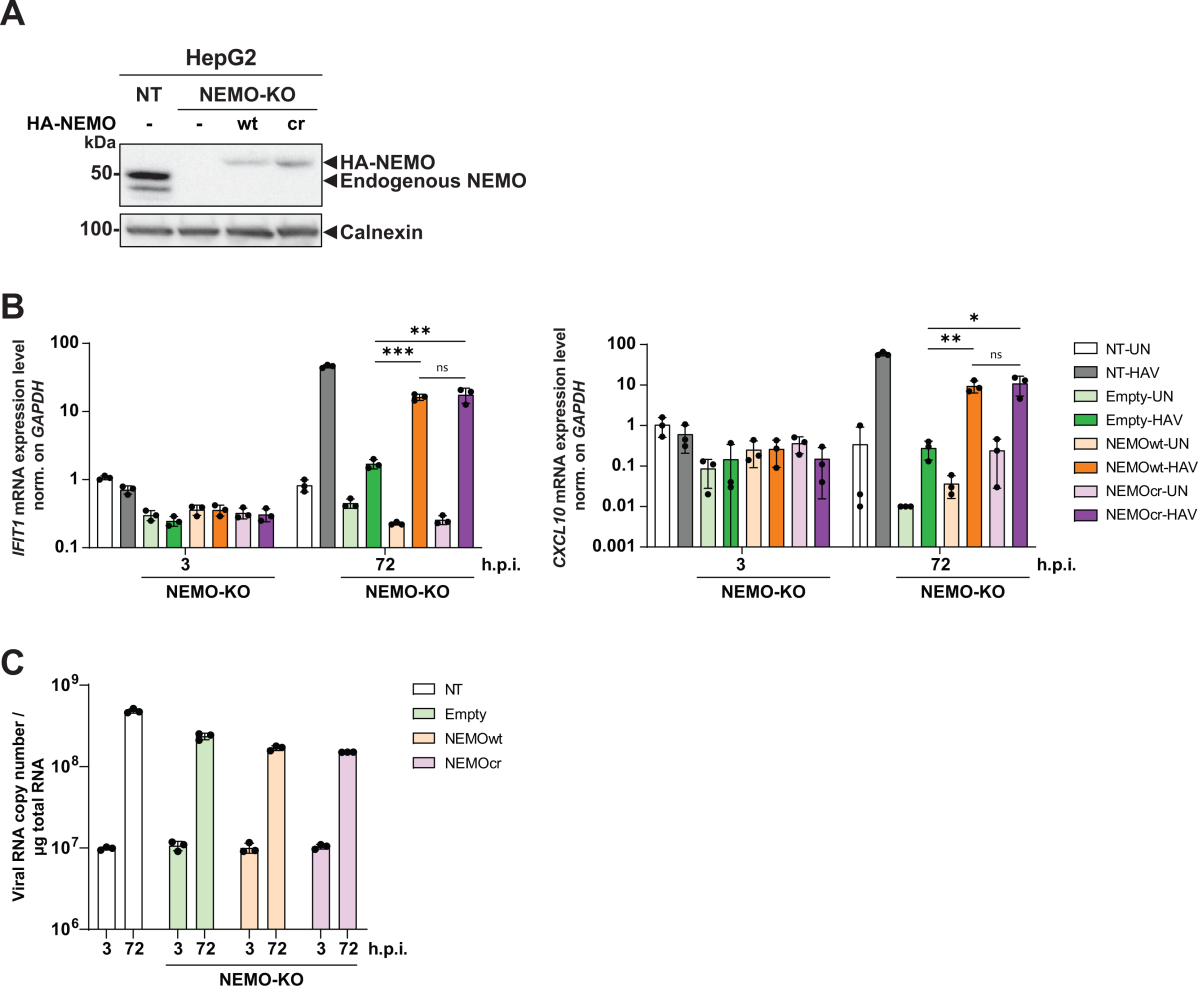
Analysis of HAV-induced innate immune response in HepG2-NEMO-KO cells reconstituted with wt and cr NEMO. (**A**) HepG2-NEMO-KO cells described in Fig. 5A were transduced with lentiviral vectors encoding a selection marker, wt, or cr HA-NEMO. Cell lysates were subjected to western blot analysis with anti-NEMO and anti-Calnexin antibodies. Calnexin was used as loading control. (**B and C**) The cells depicted in (**A**) were infected with HAV (MOI = 1.5) and harvested at the indicated timepoints for total RNA extraction and subject to *IFIT1, CXCL10* mRNA (**B**), and HAV RNA (**C**) quantification by RT-qPCR. *GAPDH* was used as a reference gene. Values were normalized to the mock control. Data represent mean ± SD of biological triplicates with technical triplicates. Statistical analysis was performed with multiple *t*-test. *, *P* < 0.05; **, *P* < 0.01; ***, *P* < 0.001. wt (wildtype); cr (cleavage-resistant); ns (non-significant); h.p.i. (h post-infection).

We next investigated the effect of NEMO-KD and -KO on HAV-induced innate immune response and HAV replication in HepG2 cells, which represent the best model to study innate immune responses upon HAV infection (17). We first determined that NEMO KD efficiency was most efficient at 48 and 72 h in uninfected cells (Fig. 6A). Subsequently, we infected HepG2 cells upon KD or KO of NEMO with HAV and quantified ISG expression, as well as HAV RNA at 3, 48, and 72 h post-infection. KD was initiated immediately after infection by transfection of siRNA to achieve sufficient NEMO reduction at the peak of HAV replication at ca. 48 h post-infection. Similar to what we observed with poly(I:C) stimulation, the NEMO-KD cells were still able to mount a full induction of *IFIT1* and *CXCL10* expression upon HAV infection, with only a slight decrease for *CXCL10* at 48 h post infection. However, as expected, KO of NEMO completely abolished ISG induction (Fig. 6B). These results suggested that NEMO cleavage by HAV did not functionally counteract ISG induction in the infection setting, not even after depletion by 80% upon knockdown. Interestingly, HAV RNA abundance was not increased upon KD or KO of NEMO (Fig. 6C), suggesting that the level of ISG induction by HAV was not sufficient to counteract replication, at least within the timeframe of these experiments. In line with the moderate ISG induction mounted by HAV (17), *TNFAIP3* expression was not upregulated upon HAV infection (Fig. 6D), probably due to the overall relatively low induction levels observed for this gene also upon poly(I:C) stimulation (Fig. 5B). To finally prove the specificity of the phenotype of the NEMO-KO cells, we reconstituted expression with HA-NEMO wt or HA-NEMO cr (Fig. 7A). If NEMO cleavage by HAV would substantially impair innate signaling, a cleavage-resistant variant was expected to increase ISG induction and counteract viral replication upon HAV infection. However, both NEMO variants rescued *IFIT1* and *CXCL10* induction to the same extent (Fig. 7B) and had no impact on viral replication (Fig. 7C).

In sum, our results suggest that HAV fails to counteract the innate immune response by proteolytic cleavage of NEMO. Since we found that NEMO was fully functional at a very low expression level in hepatocytes, limited cleavage by 3C did not appear to result in a significant disruption of innate immune response by HAV.

## DISCUSSION

This study provides an extensive investigation on the potential HAV counteraction to NEMO employing human liver-derived cell culture models. Consistent with a previous study, we found partial cleavage of NEMO by 3C and 3CD overexpression. However, the majority of NEMO degradation was unspecific, since a cleavage-resistant NEMO mutant was affected to the same extent. In physiological models, cleavage was almost undetectable and had no obvious impact on poly(I:C) or HAV-mediated innate immune induction. This was in line with our data demonstrating that in cell lines of hepatic origin, NEMO is important for IRF3-mediated ISG induction and essential for NF-κB signaling but is fully functional already at very low expression levels, rendering it a difficult target for viral interference.

The 3C or 3C-like protease of several RNA viruses were shown able to cleave NEMO, as that of foot-and-mouth disease virus (FMDV), another picornavirus, which not only cleaves but associates with NEMO to prevent the assembly of NEMO-containing complexes to disrupt several innate immune signaling pathways (37). SARS-CoV-2 3C-like protease was demonstrated to cleave NEMO at several sites as well, resulting in human brain endothelial cell death, disruption of the blood–brain barrier (38, 39), and immune counteraction (40). Porcine coronavirus 3C-like protease also cleaves NEMO to disrupt type I IFN signaling (41, 42). Norovirus 3C-like protease was reported to target NEMO, thus ablating type I IFN signaling (43). These studies underpin that NEMO is widely recognized as a regular substrate for the 3C protease in a wide range of infections by RNA viruses. A previous study also demonstrated HAV 3C-mediated cleavage of NEMO contributing to counteract innate immunity in the epithelial kidney cell line HEK293T (16). Similarly, upon ectopic expression of 3C in an OE system-based on transfection, we found that HAV 3C protease and its precursor 3CD diminished NEMO abundance by more than 50% in Huh7-Lunet cells, a widely used HCC-derived cell line (Fig. 3) but found no indication for functional significance of this cleavage in any of our models. More importantly, by using a cleavage-resistant NEMO mutant (16), we found that the majority of NEMO degradation was not due to specific 3C-mediated cleavage, but to unspecific events, dependent on the proteolytic activity of 3C/3CD. Cytotoxicity associated with 3C/3CD expression might be the most plausible driving force of this phenotype. Association of NEMO degradation with cytotoxicity further explains the lack of apparent activity of 3ABC in our study, which was found to be far less cytotoxic than 3CD expression (17). Potential cellular processes involved might be caspases activated by 3C/3CD expression or proteasomal degradation. However, since this phenotype was an artifact of overexpression and not found in any of the more physiological models like replicon cells and HAV infection, we did not put further efforts into a more thorough characterization.

Although we aimed to keep our experimental models as meaningful as possible, e.g., by focusing on cell lines of hepatic origin, each approach came with limitations. Due to the cytotoxicity of 3C protease (29, 30), it was impossible to select for cells stably expressing 3C, which would have offered a more robust readout; therefore, we had to rely on transient expression by lentiviral transduction. OE by plasmid transfection using T7-RNA-polymerase expression and the respective promoter showed the highest apparent cleavage efficiency but is by far the least physiological model and suffers from potential variations in transfection efficiency, limiting bulk analyses in cell lysates and finally was flawed by unspecific degradation of NEMO.

Stable HAV replicons in Huh7-Lunet cells, obtained through selection of HAV subgenomes associated with an antibiotic resistance (32), offer physiological expression levels of all viral nonstructural proteins, including the 3C precursors, in all cells of the culture. Here, we found no detectable NEMO cleavage and no counteraction to the type I IFN signaling as well as to the NF-κB pathway, in line with our recent study (17). Nevertheless, cytotoxicity of the 3C protease might select for relatively low replication levels, which is consistent with the limited efficiency in generating such persistent HAV replicon cell lines (32). This moderate expression still might reflect the physiological amount of 3C during HAV infection in hepatocytes.

Importantly, aiming at elucidating the critical threshold of NEMO protein expression necessary for effective functionality in innate immunity, we found that even upon successful KD in HepG2 and PH5CH cells, with only 10% to 20% of the original expression level, the cells were still able to respond to poly(I:C) and TNF-α, suggesting an intact type I IFN signaling and minimal impact on the NF-κB pathway. This phenomenon was here characterized for the first time in cell lines with hepatic origin and was previously described in murine pre-B cell clones, where the functional equivalence of immune responses remained unchanged, despite a 10-fold range in the expression of NEMO molecules per cell (22). Therefore, it would be necessary for HAV to ablate over 80% of the NEMO expression in order to counteract the type I IFN pathway. Since we did not observe such efficient cleavage in any of the models, it seems unlikely that NEMO counteraction could play a crucial role in restricting HAV-induced innate immune responses. The situation is further complicated by fluctuations in NEMO protein expression levels upon cell passaging (Fig. 6A) and a slight induction of *IKBKG* mRNA levels by HAV 3C expression/infection (Fig. S2), which might compensate NEMO cleavage to some extent. Consistent with our findings, a recent study also argued against NEMO being a critical factor for HAV in limiting the innate immunity (44).

HAV was demonstrated to be sensed by MDA5/LGP2, which interacts with MAVS and activates the downstream type I IFN signaling (17). Despite NEMO was found acting as a bridging factor between MAVS and IRFs (9), our data suggested that NEMO was more likely to be an auxiliary element in the RLR pathway in hepatocytes. In addition, our study supported a previous research showing that HAV failed to efficiently counteract the MDA5/LGP2 pathway, thus leading to establishment of IFN signaling *in vivo* (17). Moreover, it was also shown that HAV induces *CXCL10* expression in PHH and HepG2 cells (25). Previously, it was reported that HAV-induced NF-κB activation in the monkey kidney cell line FRhK-4 (45), which further indicated an inefficient counteraction of NEMO. Intriguingly, we did not observe an induction of the NF-κB pathway by HAV in our model, which could imply that the ISG induction by HAV was mainly downstream of IRF3 activation. However, further investigations are required to elucidate this discrepancy.

In summary, our results demonstrated that HAV 3C protease and its precursor 3CD could cleave NEMO when expressed at a high level but with no effective counteraction of the innate immune responses in physiological systems. On the one hand, this might be due to the inefficient cleavage of NEMO. On the other hand, we showed that NEMO could still function even upon 80%–90% reduced expression and sufficiently supported HAV-dependent ISG induction. Overall, our study emphasizes the necessity for a comprehensive analysis of HAV’s immune evasion mechanisms in physiological model systems.

## MATERIALS AND METHODS

### Cell culture

Huh7.5 was kindly gifted by Charles Rice. Huh7-Lunet, Huh7-Lunet expressing T7 polymerase (46), and Huh7-Lunet cells expressing pGem-FLuc-HAV-Blr replicon (32) have been described previously. PH5CH was a generous gift from Kunitada Shimotohno. HepG2 cells were kindly provided by Hans-Peter Dienes. HEK293T cells were described before (47). All cell lines used in this study were validated free of mycoplasma contamination. Cell lines were cultivated in complete Dulbecco’s Modified Eagle Medium (DMEM, Gibco), supplemented with 10% (v/v) fetal calf serum (FCS, Seromed), 1% (v/v) penicillin–streptomycin (P-S, Gibco), and 1% (v/v) non-essential amino acids (NAA, Gibco), and cultured at 37°C in a 5% CO_2_ incubator.

PHH was purchased from BioIVT and cultured in William’s Medium E (Gibco), supplemented with 1% (v/v) P-S (Gibco), 1% (v/v) NAA (Gibco), 1% (v/v) glutamax (Gibco), 0.2% (v/v) normocin (Invivogen), 2% (v/v) B27 (Gibco), 1% (v/v) N2 supplement (Gibco), 100 mM nicotinamide (Sigma-Aldrich), 1.25 mM N-acetylcysteine (Sigma-Aldrich), 10 µM Y27632 (Peprotech), 1 µM A83-01 (Tocris) and cultured at 37°C with 5% CO_2_.

The stable cell lines were kept in media containing selective antibiotics with the following final concentrations: blasticidin (5 µg/mL, Sigma-Aldrich), puromycin (2 µg/mL; 5 µg/mL in PH5CH, Sigma-Aldrich), and G418 (1 mg/mL, Geneticin, Gibco).

### Poly(I:C) and TNF-α stimulation

Poly(I:C) high molecular weight (HMW; Invivogen) was dissolved in sterile 1× phosphate-buffered saline (PBS) at a final concentration of 1 mg/mL. For supernatant feeding, poly(I:C) was added directly to the medium to the final concentration of 10 µg/mL. For the poly(I:C) stimulation by transfection, poly(I:C) was prepared in Opti- minimal essential medium (opti-MEM, Gibco) and mixed with the mixture of the transfection reagent Lipofectamine2000 (Life Technologies) to the final concentration of 0.1 or 0.5 µg/mL poly(I:C). The mixture was then incubated at RT for 5 min and was added dropwise to the cells. Recombinant human TNF-α protein (Abcam) was dissolved in 1× PBS at a final stock concentration of 0.1 µg/µL. The final concentration of 10 or 20 ng/mL TNF-α was prepared in DMEM (Gibco) and added to the cells. Cells were harvested for RNA extraction after 6 h of stimulation.

### Viral production and infection

In this study, cells were infected with the cytopathic HM175/18f strain of HAV. The HAV full-length genome RNA transcript was generated by *in vitro* transcription of a linearized plasmid encoding HAV/18f (kindly gifted by Stanley Lemon). The RNA was then delivered into Huh7.5 cells by electroporation. After 11 days, the cell lysates were harvested and filtered through 0.45 µm filters to remove cell debris.

Huh7.5 and Huh7-Lunet cells were infected at a multiplicity of infection (MOI) of 1.4 and HepG2 cells were infected at an MOI of 0.4 for 96 h or of 1 or 1.5 for 72 h.

### Plasmids

The HAV replicon plasmid was described before (32) and was kindly provided by Yuri Kusov. The HAV-18f plasmid used for HAV viral production was a kind gift from Stanley Lemon.

For HAV 3C wt, the sequence was amplified from HAV HM175/18f genome, and for the 3C mutant, the sequence was amplified from a plasmid encoding 3ABC mutant, previously generated in our laboratory (17), using primers #1 and #2 with an N-terminal Flag-tag. 3CD wt, 3CD mutant, 3ABC wt, and 3ABC mutant were generated previously in our laboratory (17). For NEMO-encoding gene, the *IKBKG* sequence was amplified from a cDNA bacteria stock (ORFeome library) in the Department of Infectious Diseases, Molecular Virology, Heidelberg University using primers with and HA-tag at the N-terminal (#3 and #6) or without (#4 and #6). For NEMO cr, the cleavage site Q304 was reported previously (16), and the resistant mutation (Q304A) was introduced by overlap extension PCR using primers (#7 and #8). For cloning into the pWPI-ROSA plasmid, primers used for *IKBKG* genes are #5 and #6 and to increase detection sensitivity, two consecutive HA-tags were constructed at the N-terminal of *IKBKG*. The primers used for amplification are listed in Table 1. The PCR amplification was carried out using Phusion Flash High-Fidelity PCR Master Mix (ThermoFisher Scientific) according to manufacturer’s guide. The inserts then were cloned into the pTM-1.2 vector (48), which was used for overexpression experiments described in reference 49 or pWPI vectors for lentiviral vector production.

**TABLE 1:** Primers for cloning plasmids

Primer no.	Sequence (5′→3′)
1	fwd-gatccccggggatcatggactacaaagacgatgacgacaagtcaactttggaaatagcaggact
2	rev-gatcactagtgatcctactgactttcaattttcttatcaatattttgg
3	fwd-gatcggatccgatcatgaataggcacctctggaagag
4	fwd-gatcggatccgatcatgtatccctatgacgtccccgactacgcggccatgaataggcacctctggaagag
5	fwd-gatcgcgatcgcgatcatgtatccctatgacgtccccgactacgcggccatgaataggcacctctggaagag
6	rev-gatcactagtgatcctactactcaatgcactccatgacat
7	fwd-ccggtgctgaaggccgcagcggatatctacaag
8	rev-cttgtagatatccgctgcggccttcagcaccgg
9	fwd-caccgattctcctccaggcagcgc
10	rev-aaacgcgctgcctggaggagaatc

For generating LentiCRISPRv2-NEMO-KO plasmid, the CRISPR sgRNA primers (#9 and #10) were designed to target exon 2 of *IKBKG* (NCBI reference sequence: NM_003639.4) using the website (http://www.e-crisp.org/) described in reference 50. The sgRNA were first dimerized with the T4 polynucleotide kinase (PNK) and were ligated into the vector LentiCRISPRv2_Puro.

### DNA transfection of pTM plasmids

Huh7-Lunet-T7 cells were seeded 1.5 × 10^5^ in a six-well plate 1 day before transfection. Prior to the transfection, the medium was replaced with 750 µL FCS-depleted DMEM. Then, 2.5 µg of the plasmid mixture was mixed with 7.5 µL of TransIT-LT1 Reagent (Mirus Bio LLC) in a total of 250 µL Opti-MEM and was incubated at room temperature (RT) for 20 min and was added dropwise to the cells. Cells transfected with pTM-GFP were used as a control and the transfection efficiency were checked 24 h after transfection. The transfection efficiency was approximately 90%, and cells were harvested after 24 h of transfection.

### Western blot

Cells were harvested and lysed in the lysis buffer (50 mM Tris/HCl pH 7.4; 150 mM NaCl; 1% Triton X-100), supplemented with protease inhibitor cocktail (Roche), and incubated on ice for at least 30 min with vortexing every 10 min. The cell lysates were centrifuged at 14,000 rpm for 15 min, and the supernatant was collected for Bradford assay using Protein Assay Dye Reagent Concentrate (Bio-Rad), and each sample was adjusted to the same protein concentration. The supernatant was mixed with 6× Laemmli buffer (0.375M Tris-HCl, 9% SDS, 50% Glycerol, 0.03% Bromophenol Blue, 9% β-mercaptoethanol), boiled at 95°C for 5 min. Samples were loaded to 12% sodium dodecyl sulfate-polyacrylamide gel electrophoresis (SDS-PAGE) gels for protein separation and were blotted on polyvinylidene difluoride (PVDF) membranes (Merk Millipore). Membranes were then blocked with 5% milk/0.15% Tween-20 in PBS (PBS-T) for 1 h and incubated in 5% milk/PBS-T overnight at 4°C with primary antibodies against NEMO (rabbit; 1:1,000; Cell signaling; # 2685S), Calnexin (rabbit; 1:2,000–1:1,000; Enzo Life Sciences; # ADI-SPA-860-F), HAV 3C protease (rabbit; 1:5000 ; kindly gifted by Dr. Yuri Kusov, Medical University of Lübeck, described in (51)), HA (mouse; 1:10,000; Sigma-Aldrich; # H3663-200UL), and Flag (rabbit; 1:4,000; Sigma-Aldrich; # F7425-.2MG). Membranes were then washed with PBS-T for three times and incubated with secondary antibodies (1:10,000 for anti-mouse and 1:5,000 for anti-rabbit secondary antibody) for 50 min. After membranes were washed with PBS-T for three times, chemiluminescence was detected using the enhanced chemiluminescence (ECL) reagent, Western Lightning Plus-ECL (Revvity Health Sciences), or SuperSignal West Femto Maximum Sensitivity Substrate (Thermo Scientific). The imaging data were acquired by the ChemoStar Professional (Intas Science Imaging) software. Quantification of the image was measured with the Fiji software.

### Lentiviral vectors production and transduction

Approximately 5 × 10^6^ HEK293T cells were seeded in 10 cm dishes 1 day prior to transfection. Before transfection, the medium was replaced with 5 mL of FCS-depleted DMEM. The plasmid mixture was prepared with 5.14 µg of the plasmid pSPAX2-Gag-Pol, 1.71 µg of the plasmid pMD2-VSVG, and 5.14 µg of pWPI plasmids encoding genes of our interests (ratio 3:1:3) in 400 µL of Opti-MEM. The plasmid mixture was then mixed with 36 µL of polyethylenimine (PEI, 1 mg/mL, Polysciences Inc) in 400 µL of Opti-MEM. The mixture was incubated at RT for 20 min and added dropwise to HEK293T cells. Medium was changed to 8 mL complete DMEM after 6 h. At 48 and 72 h after transfection, the supernatant was harvested and filtered with 0.45 µm filters to remove cell debris.

For generating stable cell lines, 1.5 × 10^5^ of Huh7.5 cells were seeded in a six-well plate. At 16, 24–28, and 48 h after seeding, the medium was replaced with 1 mL of the lentiviral supernatant. At 4 h after the last transduction, the medium was changed to complete DMEM, and on the next day, the medium with the selective antibiotics was added to the cells for selection.

For establishing transient protein expression in the cells, 9 × 10^4^ Huh7.5 or Huh7-Lunet cells were seeded in a 12-well plate. At 16, 24–28, and 48 h after seeding, the medium was replaced with 150 µL of the lentiviral supernatant. At 4 h after the last transduction, the medium was changed to complete DMEM. On the next day, the cells were then subjected to treatment of indicated stimuli.

### RNA extraction and quantitative reverse transcription polymerase chain reaction (RT-qPCR)

The total RNA was extracted using the NucleoSpin RNA Plus kit (Macherey-Nagel). RT-qPCR was performed as described before (52). Complementary DNA (cDNA) was generated from the RNA using the High-Capacity cDNA Reverse Transcription Kit (ThermoFisher Scientific). qPCR was performed using 2× iTaq Universal SYBR Green Supermix (Bio-Rad), and the reactions were carried out on a CFX96 Touch real-time PCR detection system (Bio-Rad) with the following protocol: 95°C for 3 min, 95°C for 10 s, and 60°C for 30 s.

For detecting HAV RNA, one-step RT-qPCR was performed using qScript XLT One-Step RT-qPCR ToughMix (Quanta Biosciences). A serial dilution of RNA standards (10^2^ to 10^9^ HAV RNA copies) was performed in parallel to determine the absolute RNA amounts. Specific primers for HAV-IRES and HAV probe (TGTTAAGACAAAAACCAATTCAACGCCGGA) were used. Reactions were carried out with the following protocol: 50°C for 10 min, 95°C for 1 min, and 40 cycles as follows: 95°C for 10 s, 60°C for 1 min. All primers used are listed in Table 2. To compare *IKBKG* expression in different cell lines, the mRNA level of *IKBKG* was normalized on the geometric mean of two housekeeping genes, *GAPDH* and *HPRT* (27).

**TABLE 2:** Primers for RT-qPCR

Gene name	Sequence
*IKBKG*	fwd- AGCACCTGAAGAGATGCCAGCA rev- AGCCTGGCATTCCTTAGTGGCA
*IFIT1*	fwd- GAAGCAGGCAATCACAGAAA rev- TGAAACCGACCATAGTGGAA
*CXCL10*	fwd- GGCATTCAAGGAGTACCTCTCTC rev- TGGACAAAATTGGCTTGCAGGA
*TNFAIP3*	fwd- TCCTCAGGCTTTGTATTTGAGC rev- TGTGTATCGGTGCATGGTTTTA
*GAPDH*	fwd- GAAGGTGAAGGTCGGAGTC rev- GAAGATGGTGATGGGATTTC
*HPRT*	fwd- GCGTCGTGATTAGCGATGATG rev- CTCGAGCAAGTCTTTCAGTCC
HAV-IRES	fwd- GGTAACAGCGGCGGATATTGG rev- AGTCAATCCACTCAATGCATCCA

### Cell viability assay

Approximately, 7 × 10^3^ Huh7.5 cells were seeded in a 96-well plate. On the next day, the medium was changed to FCS-free DMEM before transduction. Cells were transduced for three times at 16, 24–28, and 48 h after seeding with 100 µL of lentiviral vectors. At 48 h (48 h post-transduction experiment) or 96 h (96 h post-transduction experiment) after the first transduction, medium was removed, and 30 µL of the CellTiter-Glo 2.0 reagent (pre-diluted 1:2 in 1× PBS, Promega) was applied onto the cells. Luminescence was measured at OD560 using the plate-reader software Microwin2000 version 4.41 with the measurement settings of “Shake: 10 s, fast” and “Delay: 1 s”.

### Immunofluorescence assay

Approximately 4 × 10^4^ (Huh7.5 and Huh7-Lunet) or 5 × 10^5^ (HepG2) cells were seeded in the 24-well plates. Cells were fixed with 4% paraformaldehyde (PFA) in 1× PBS for 15–20 min at room temperature and were washed with 1× PBS for three times. Cells were permeabilized with digitonin (diluted 1:2,000 in 1× PBS) for 15 min, and the cells were washed with 1× PBS for three times. Cells were then blocked with 3% BSA in 1× PBS for 30 min at room temperature. The primary antibodies, anti-Flag (mouse; 1:100; Sigma-Aldrich; # F1804-.2MG) and anti-HAV-VP3 (mouse; 1:50; Thermo Fisher Scientific; # MA1-7371), were diluted in 3% BSA/1× PBS and incubated with the cells overnight at 4°C. Cells were then washed with 1× PBS for three times. After washing, coverslips were stained with secondary antibody and 4’,6-diamidino-2-phenylindole (DAPI) (1:1,000; Invitrogen) for 45 min at room temperature in the dark. Secondary antibody, anti-mouse IgG2a-AlexaFluor488, was diluted 1:1,000 in 3% BSA/1× PBS . Cells were then washed with 1× PBS for three times. Coverslips were mounted by using Fluoromount G Reagent (Southern Biotech). Fluorescent signals were acquired with a Nikon Ti Eclipse epifluorescence microscope using a 40× oil immersion objective and analyzed with the NIS-Element AR software package. Images were analyzed and merged with the Fiji software.

### siRNA transfection

Approximately 7 × 10^4^ PH5CH cells or 3 × 10^5^/well (HepG2) were seeded in a 12-well plate 1 day prior to the transfection. The non-targeting and *IKBKG* siRNA SMARTpool (5 nmol, Horizon Discovery) were dissolved in RNase-free water to a final concentration of 20 µM. Before transfection, the medium was changed with 400 µL FCS-free DMEM. For each well, 0.75 µL of siRNA was prepared in 75 µL Opti-MEM and mixed with 4.5 µL Lipofectamine RNAiMAX (Thermo Fisher Scientific) in 75 µL Opti-MEM. The mixture was incubated at RT for 5 min, and a total of 150 µL mixture was added to the cells dropwise. After 48 h of transfection, the cells were either treated with poly(I:C), TNF-α, or infected with HAV.

### CRISPR-Cas9 KO clones

Approximately, 1.5 × 10^5^ of PH5CH and 5 × 10^5^ of HepG2 cells were seeded in a six-well plate. The cells were transduced with lentiviral vectors encoding Cas9 and non-target or *IKBKG*-specific sgRNA (see “Plasmids”). On the next day, medium was changed into complete DMEM, and 1 day later, the selective antibiotic puromycin (2 µg/mL for HepG2; 5 µg/mL in PH5CH) was added to the cells for selection. Selection was done in 3–5 days, followed by the validation of the KO pools by Western blot. The KO cell pool was further seeded for single-cell clone selection in 96-well plates for 2–3 weeks. After selection, phenotypes of KO clones were validated by Western blot.

### Statistics

Statistical analysis was performed by multiple unpaired *t*-test or Welch’s unpaired *t*-test using GraphPad Prism 8 software (GraphPad Software, La Jolla, CA, USA). *P < 0.05; **P < 0.01; ***P < 0.001; ****P < 0.0001.

## Data Availability

All data supporting the findings of this study are included within the article and its supplemental material.

## SUPPLEMENTAL MATERIAL

Fig. S1 and S2 jvi.02264-24-s0001.docx HAV 3C/3CD counteraction by NEMO degradation and impact of HAV on NEMO mRNA levels.

ASM does not own the copyrights to Supplemental Material that may be linked to, or accessed through, an article. The authors have granted ASM a non-exclusive, world-wide license to publish the Supplemental Material files. Please contact the corresponding author directly for reuse.
