# Supplementary material for: Limited impact of hepatitis A virus 3C protease-mediated cleavage on the functions of NEMO in human hepatocytes
Source: J Virol. 2025 Jan 24;99(2):e02264-24. doi: 10.1128/jvi.02264-24 (PMC11852894; doi:10.1128/jvi.02264-24)
Supplement: Fig. S1 and S2 — HAV 3C/3CD counteraction by NEMO degradation and impact of HAV on NEMO mRNA levels. [file jvi.02264-24-s0001.docx]

**SUPPLEMENTAL MATERIAL**

**Limited impact of hepatitis A virus 3C protease-mediated cleavage on the functions of NEMO in hepatocytes**

Hao-En Huang, Ombretta Colasanti, Teng-Feng Li, Volker Lohmann

**Contents:**

Supplemental Figures 1-2

**SUPPLEMENTAL FIGURES**

**Figure S1**

**
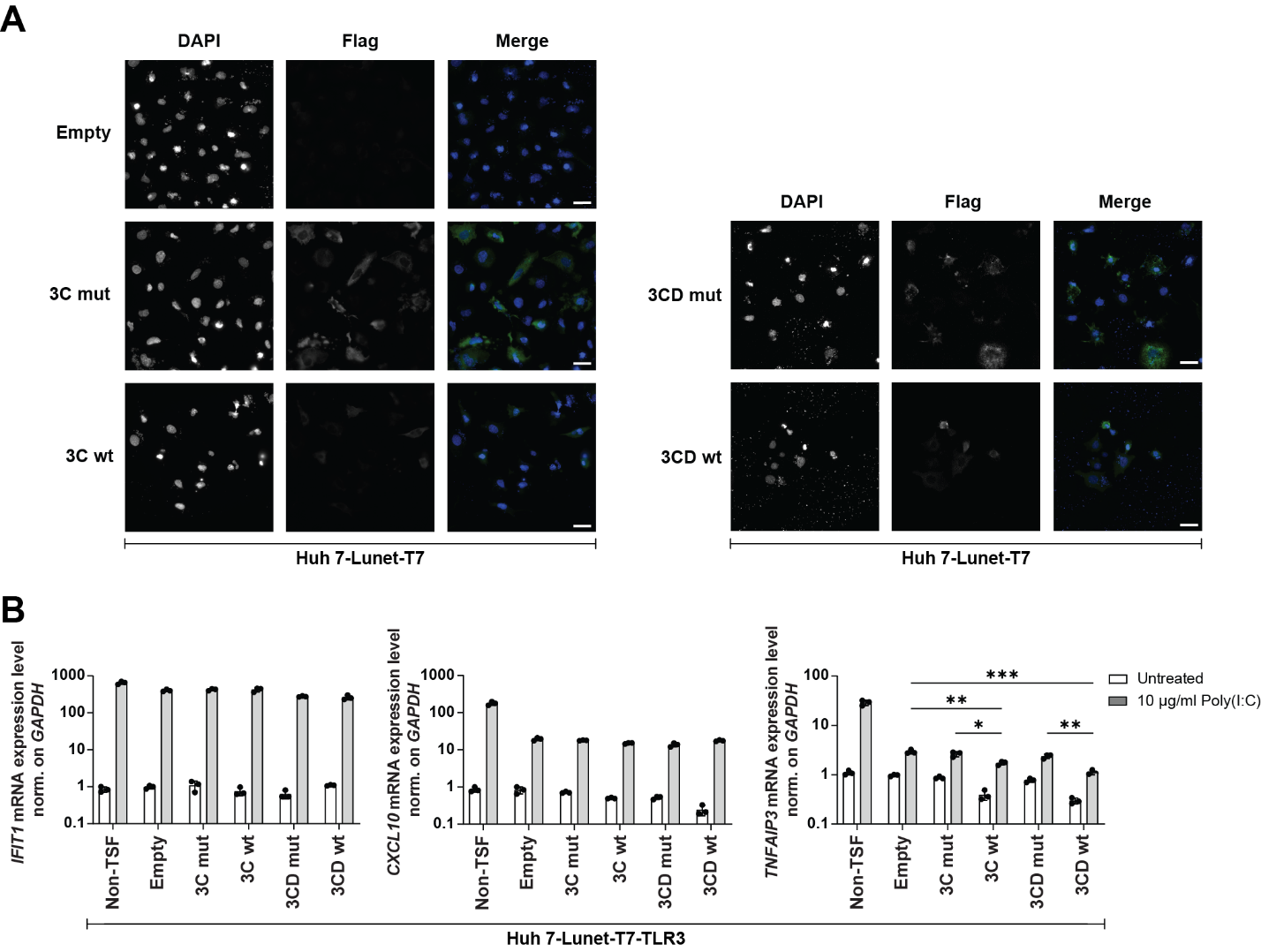
**

**Supplemental Figure 1. Functional analysis of HAV protease counteraction of NEMO in Huh7-Lunet-T7-TLR3 cells.** (A) Huh7-Lunet-T7 cells were transfected with an empty pTM plasmid or the ones encoding Flag-3C mut, Flag-3C wt, Flag-3CD mut, and Flag-3CD wt (1.5 μg) for 24 h. Cells were stained with anti-Flag (green). Nuclei were stained with DAPI. Images were taken at 40X. Scale bar, 40 μm. (B) Huh7-Lunet-T7 cells were reconstituted with TLR3 by lentiviral transduction. The cells were then left untreated or transfected (TSF) as described in (A) for 12 h. After stimulation with 10 μg/ml poly(I:C) or left untreated for 6 h, total RNA was isolated, and the respective mRNA levels were quantified by RT-qPCR. *GAPDH* was used as a reference gene. Values were normalized to the empty control. Note that even transfection of empty vector reduced induction of CLX10 and TNFAIP3, compared to Non-TSF controls. Data represent mean ± SD of biological triplicates with technical triplicates. Statistical analysis was performed with multiple t-test. *, p < 0.05; **, p < 0.01; ***, p < 0.001. mut (mutant); wt (wildtype).

**Figure S2**


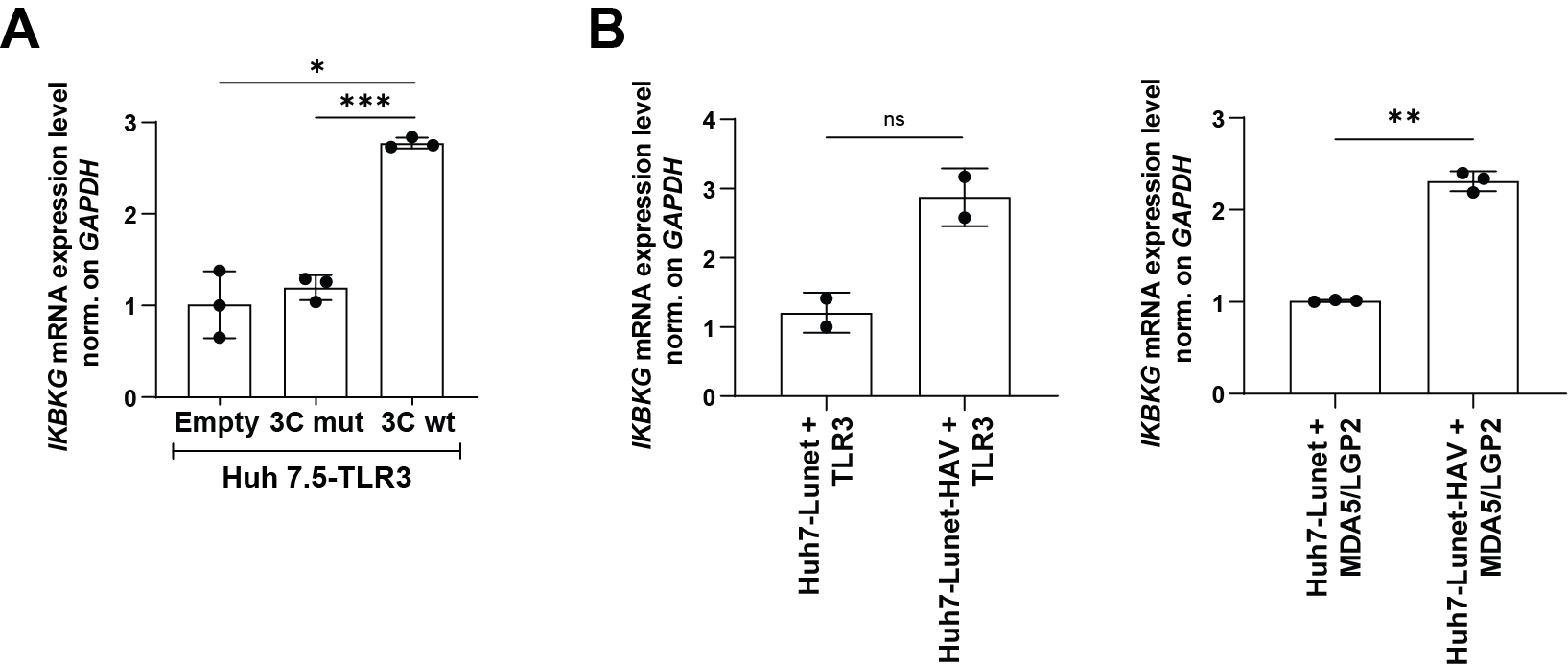


**Supplemental Figure 2. Impact of HAV 3C and HAV replication on *IKBKG* mRNA expression.** (A) Huh7.5-TLR3 cells were transiently transduced with lentiviral vectors encoding a selection marker (Empty), Flag-3C mut, or Flag-3C wt. (B) Huh7-Lunet naïve cells and cells expressing HAV replicons were transiently transduced with the lentiviral vectors encoding TLR3 (left panel) or MDA5 and LGP2 (right panel). Total RNA was isolated, and the *IKBKG* mRNA levels were quantified by RT-qPCR. *GAPDH* was used as a reference gene. Values were normalized to the empty control. Data represent mean ± SD of biological duplicates or triplicates with technical triplicates. Statistical analysis was performed with multiple t-test. *, p < 0.05; **, p < 0.01; ***, p < 0.001. mut (mutant); wt (wildtype).
